# Detection of lung allograft injury through a comprehensive multidisciplinary analysis of donor-derived cell-free DNA in plasma and bronchoalveolar lavage: a real-world single center experience

**DOI:** 10.3389/fimmu.2025.1619771

**Published:** 2025-09-05

**Authors:** Fiorella Calabrese, Federica Pezzuto, Luca Vedovelli, Cecilia De Chellis, Francesca Lunardi, Monica Loy, Eleonora Faccioli, Marta Vadori, Davide Biondini, Serena Marinello, Fausto Braccioni, Federica Meloni, Marco Schiavon, Chiara Giraudo, Claudia Del Vecchio, Deborah J. Levine, Emanuele Cozzi, Federico Rea

**Affiliations:** ^1^ Department of Cardiac, Thoracic, Vascular Sciences and Public Health, University of Padova, Padova, Italy; ^2^ University Hospital of Padova, Padova, Italy; ^3^ Department of Molecular Medicine, University of Padova, Padova, Italy; ^4^ Division of Pulmonary, Critical Care and Allergy, Stanford University, Palo Alto, CA, United States

**Keywords:** donor-derived cell-free DNA (dd-cfDNA), lung transplantation, allograft injury, bronchoalveolar lavage (BAL), plasma biomarkers

## Abstract

**Introduction:**

Plasma donor-derived cell-free DNA (dd-cfDNA) is an emerging potential tool for diagnosing lung graft injury. This study explored the relevance of dd-cfDNA levels in different graft injuries thoroughly characterized after a well-established multidisciplinary team approach. The usefulness of bronchoalveolar lavage (BAL) dd-cfDNA in complementing detection of allograft injury was also investigated.

**Methods:**

Plasma dd-cfDNA was measured by next generation sequence on 127 samples from patients visited consecutively, contemporaneously with a systematic analysis of surveillance transbronchial biopsy by LASHA template, BAL analysis and immunological monitoring.

**Results:**

Patients with immunological injury exhibited the highest plasma dd-cfDNA levels (median 2.67%), with a sensitivity of 100% while patients with non-immunological insults showed a sensitivity of 28%. The combination of BAL with plasma dd-cfDNA improved the sensitivity for detecting non-immunological injury from 28% to 71%. Random forest analysis showed that plasma dd-cfDNA >1% was among the most important variables in predicting death and chronic lung allograft dysfunction.

**Discussion:**

Our data suggests that plasma dd-cfDNA is a useful tool for immunological graft injury assessment. The performance of BAL dd-cf DNA needs to be validated on larger case series. The integration of plasma dd-cfDNA with other post-transplant follow-up investigations may allow more sensitive diagnoses and appropriate graft injury management.

## 1 Introduction

Lung transplantation offers a potentially curative option for patients with types of end-stage lung diseases, providing hope when conventional medical therapies have been exhausted.

In recent years the number of lung transplants has increased significantly with over 4,000 procedures performed annually (1). Lung transplant patients face higher rates of immunological and non-immunological complications, and long-term outcomes remain less favorable compared to other solid organ transplants. Close monitoring of recipients is essential to detect early signs of graft dysfunction. Current approaches for evaluation of allograft injury include clinical assessment, pulmonary function testing (PFT), imaging, measurements of donor specific antibodies (DSAs) and bronchoscopy with bronchoalveolar lavage (BAL) and transbronchial biopsy (TBB). Given the limitations of these traditional testing methods, teams integrate results of these studies to assess graft health. Recent studies highlight donor-derived cell-free DNA (dd-cfDNA), a plasma molecular biomarker, as a promising indicator of graft injury in solid organ transplantation (2–4).

In the lung transplant setting, dd-cfDNA has been primarily explored as a diagnostic adjunct in the detection of acute rejection, particularly antibody-mediated rejection (AMR), with several studies demonstrating elevated levels in association with alloimmune injury (2, 5, 6). However, growing evidence suggests that dd-cfDNA is a general marker of tissue injury and cell turnover, and its elevation may also occur in non-alloimmune contexts, such as infection or ischemia-reperfusion damage (7–9). Despite these insights, the role of dd-cfDNA in lung transplantation remains less defined than in kidney or heart transplantation. Most published studies are limited by retrospective design, small cohorts, or lack of standardized injury classification. Furthermore, only few studies have integrated dd-cfDNA with structured histopathological assessment or addressed the potential value of dd-cfDNA in BAL fluid, a compartment potentially more reflective of localized lung injury (10, 11).

The primary aim was to assess the clinical relevance of dd-cfDNA levels across various graft injuries thoroughly characterized and discussed within a well-established multidisciplinary team (MDT) framework. A second objective was to evaluate the potential of BAL dd-cfDNA in complementing detection of allograft injury.

## 2 Materials and methods

### 2.1 Patient clinical data

This is a single-center prospective study performed on 100 bilateral lung transplant recipients who consecutively underwent scheduled monitoring after surgery from October 2022 to October 2024 at the University Hospital of Padova. All patients underwent routine post-transplant monitoring with regular clinical visits, spirometry, imaging, surveillance bronchoscopy with BAL and TBB, and DSA measurements. All BAL and plasma samples analyzed in this study were collected exclusively during these scheduled surveillance visits. No samples were obtained during clinically indicated procedures prompted by acute clinical symptoms, spirometric decline, or radiologic abnormalities.

**Figure 1:**
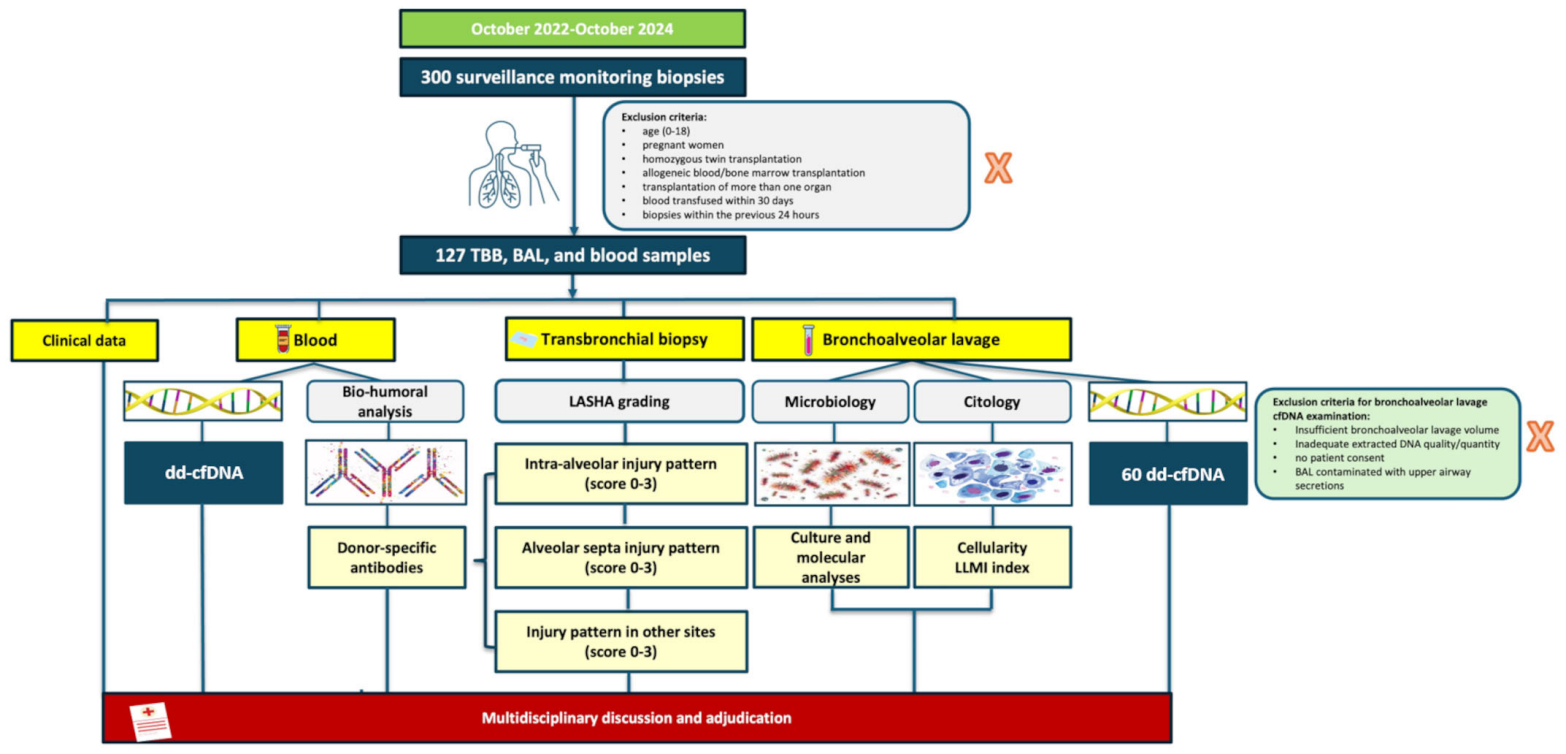
Consort diagram.

At our center, surveillance protocol includes scheduled medical appointments as previously reported (12) and cause evaluations in case of respiratory exacerbations. Precise inclusion/exclusion criteria are reported in **Figure 1**. The “Strengthening the Reporting of Observational Studies in Epidemiology” (STROBE) statement guidelines for reporting observational studies were followed. All patients provided informed consent, and the study was approved by our Institutional Review Board.

### 2.2 Microbiological and anti-HLA antibody screening

Plasma and BAL samples underwent standard culture evaluations for bacteria, mycobacteria, and fungi, including molecular assessments for viruses and non-cultivable bacteria. Infection was defined according to the International Society for Heart Transplant (ISHLT) guidelines as the detection of a clinically significant organism in microbial cultures, accompanied by mucopurulent discharge observed during bronchoscopy or focal radiographic abnormalities on thoracic imaging. Additionally, an inflammatory response, demonstrated by lymphocytosis and/or neutrophilia on cytological evaluation, was required to support the diagnosis (13). In contrast, colonization was defined as the presence of microorganisms detected on microbial cultures (10^4^ colony-forming unit - CFU) without evidence of mucopurulent discharge, focal radiographic abnormalities, or an associated inflammatory response, indicating a non-pathogenic or commensal state without active disease.

Anti-HLA antibodies were evaluated at the time of the reference biopsy. Anti-HLA IgG reactivity was analyzed with bead-based assays using the Werfen, Single Antigen Bead kits (Waukesha Wi, USA). Single antigen results with a mean fluorescence intensity (MFI) greater than 500 were considered as positive.

### 2.3 Histopathology and cytological analysis

The histopathologic evaluation of TBB specimens was performed according to the revised ISHLT working group recommendations and the LASHA template (12). Morphological features suggestive of antibody mediated rejection (AMR) were systematically reported in accordance with LASHA and further discussed in MDT meetings. The certainty of AMR was determined based on the ISHLT consensus statement (14).

### 2.4 Final diagnosis after MDT

At our center a MDT meets weekly to review and discuss patient data, ultimately reaching a consensus on the diagnosis of the observed graft lesion to guide appropriate clinical management. The MDT report includes a morphological description of the biopsy (according to LASHA), humoral and infectious data, imaging findings and cytological information of the BAL. All MDT reports for enrolled patients were independently adjudicated by a predefined expert panel. This panel included two pulmonologists (FM, FB), one immunologist (EC), one radiologist (CG), and two pathologists (FC, FP), and was further supported by an external expert in lung transplantation and antibody-mediated rejection (DL, Stanford University), who provided additional oversight and expertise in complex cases. Four categories were identified: 1) No injury (group 1), 2) Immunological injury: including acute cellular rejection (ACR), AMR, and Chronic Lung Allograft Dysfunction (CLAD) (group 2), 3) non-immunological injury: including infections or gastroesophageal reflux disease (GERD)-lung aspiration (group 3) and 4) Mixed immunological and non-immunological injury (group 4).

Cases with ACR<1 without allograft dysfunction and possible subclinical AMR were included in group 1. The MDT was blinded to dd-cfDNA% data.

### 2.5 dd-cfDNA collection, processing, and analysis

Plasma and BAL samples were collected using Streck Cell-Free DNA tubes and processed within two hours of collection. After centrifugation, aliquots were stored at −80°C. Extraction of cfDNA was performed using the QIAamp MinElute ccfDNA kit (QIAGEN, Venlo, Netherlands), and downstream analysis was conducted using the AlloSeq^®^ cfDNA kit (CareDx, CA, USA). Next-generation sequencing (NGS) was performed on the Illumina MiniSeq 3000 platform (2 × 150 bp). Library preparation, single nucleotide polymorphism (SNP) genotyping, and paired-end read mapping were used to quantify the proportion of dd-cfDNA, expressed as the percentage of donor-specific SNPs among total SNPs. The software and quality control pipelines provided by CareDx were used to determine dd-cfDNA percentages. For plasma, the 1% threshold was predefined by the manufacturer and supported by previous literature as the clinically relevant cut-off for detecting allograft injury in solid organ transplantation. For BAL fluid, where no established literature standard exists, a cut-off of 10% was defined based on cross-validated ROC analysis and supported by a subset of pre-/post-transplant paired controls. Additionally, we performed *post-hoc* analyses using alternative thresholds to assess how test characteristics varied across cutoffs; results consistently supported the selection of the 10% value (see **Supplementary Methods** for detailed rationale and performance metrics). This threshold was derived from the average of fold-specific Youden index optima in a 10-fold cross-validation framework (mean ≈ 9.7%) and rounded to 10% for clinical interpretability.

For further technical details, including pre-analytic handling, sequencing metrics, SNP filtering, and validation of BAL-specific dd-cfDNA quantification, please refer to the **Supplementary Material**.

Plasma samples were collected concurrently with TBBs in all patients. Additionally, a subset of patients underwent repeated plasma sampling for dd-cfDNA during their scheduled visits. In a subset of patients, aliquots of BAL were collected for this analysis.

### 2.6 Data integration and statistical analysis

All assessment data were meticulously recorded within the dedicated REDCap platform.

Continuous variables were expressed as median (interquartile range), and categorical variables as counts (percentages). Mann-Whitney, Pearson’s Chi-squared test, Wilcoxon rank sum test, and Fisher’s exact test were employed as appropriate. Correction for multiple comparisons was made by adjusting the false discovery rate (FDR) to control the type I error rate.

#### 2.6.1 Receiver operating characteristic curves and cut-offs

Predictive models were evaluated through cross-validation to ensure the robustness and generalizability of the results.

#### 2.6.2 Linear regression analysis

To explore the factors associated with the percentage of dd-cfDNA in plasma, we performed a multivariable linear regression analysis. The outcome variable was the dd-cfDNA plasma percentage, and the independent variables were selected based on clinical relevance and prior literature. The regression model included time from lung transplant in months, recipient sex, and the type of graft dysfunction. Additional variables included recipient age in years, donor sex, donor cause of death, donor age in years, and donor marginal status.

#### 2.6.3 Time-to-event analysis

Time-to-event graphics were obtained with the method of Kaplan-Meier (**Supplementary Data**, **Supplementary Figure 1**). P-values and univariate regressions were calculated using Cox proportional hazards regression.

#### 2.6.4 Variables selection

Random forests were used to identify and rank variables associated with death and CLAD. The method was chosen for its robustness to multicollinearity, non-linearity, and interactions among predictors (**Supplementary Table 1**).

More details regarding microbiological/immunological screening, histological/cytological analysis, detection of dd-cfDNA in plasma samples and BAL, data interpretation and statistical analyses are available in the **Supplementary Material**.

## 3 Results

### 3.1 Patient characteristics and categorization of post-transplant complications

Among 300 Caucasian recipients regularly visited at our center between October 2022 to October 2024, 100 patients were consecutively enrolled following the exclusion/inclusion criteria (**Figure 1** **);** 61% were males, with a median age of 50 years (IQR 40–63). The median time from transplantation to the collection of plasma samples was 10 months (IQR 3–45 months). Major demographic characteristics are reported in **Table 1**. As for graft injury, 52% of the cohort showed some form of graft damage: 15% categorized as *Group 2* (immunological injury), 28% as *Group 3* (non-immunological injury) and 9% as *Group 4* (both immunological and non-immunological injury). Among the patients in the immunological group, one was graded as ACR (grade A3), four as AMR (3 possible, 1 definite), and 10 CLAD (1 restrictive, 7 obstructive, 2 mixed; one with concurrent AMR). In AMR cases, the most frequently observed histological lesions included severe septal widening, moderate/severe granulocyte septal infiltration, presence of hyaline membranes, septal or intra-alveolar granulation tissue plugs (organizing pneumonia)/pneumocyte hypertrophy, and high scores of hemosiderophages. The remaining 48% of patients showed no signs of graft injury (*Group 1*) **Table 2**.

**Table 1:** Demographic characteristics of the study population.

Characteristics	N (%) or median (Q1,Q3)
Recipient characteristics	
Sex	
*Males*	61 (61%)
*Females*	39 (39%)
Age (years)	50 (40,63)
Time from lung transplant to sample collection (months)	10 (3,45)
Native disease	
Obstructive*	40 (40%)
Restrictive**	42 (42%)
Other***	18 (18%)
Donor characteristics	
Sex	
*Males*	58 (58%)
*Females*	42 (42%)
Age (years)	47 (29,56)
Expanded**** criteria donor	
*Yes*	40 (40%)
*No*	60 (60%)

_*_ Bronchiectasis, chronic obstructive pulmonary disease, cystic fibrosis.

_**_ Hypersensitivity pneumonitis, Idiopathic pulmonary fibrosis.

_***_ Pulmonary vascular diseases.

_****_Expanded Donor Criteria for Lung Transplantation, including: Age >55–60 years, smoking history (>20 pack-years or active smoker), PaO_2_/FiO_2_ <300 but >200 mmHg, resolved pulmonary infections (negative cultures), lung contusions or trauma, radiological abnormalities (clinically insignificant, unimportant or resolving), prolonged ventilation (>72–96 hours), positive serology (e.g., Cytomegalovirus, Hepatitis B Virus, Hepatitis C Virus), single-lung use (lung split), history of low-risk remote neoplasia, anatomical size mismatch (manageable), and *ex* vivo lung perfusion.

**Table 2:** Categorization of post-transplant complications and associations with plasma dd-cf DNA.

Categorization	N°	dd-cfDNA values (median,IQR)
No injury	48 (48%)	0.39 (0.22,0.61)
Immunological injury	15 (15%)	2.67 (1.38,4.06)
Non-immunological injury	28 (28%)	0.68 (0.34,1.15)
Mixed immunological and non-immunological injury	9 (9%)	3.39 (1.39,3.93)

Anti-HLA antibody testing was available for the entire cohort. Prior to transplantation DSA were not observed in 79% of cases whilst 21% had non-donor-specific anti-HLA antibodies. Following transplantation *de novo* DSA were detected in only 7% of cases. A complete dataset with typing for 4 HLA loci (HLA A, B, DRB1 and DQB1) was available for 62 cases. Among the 62 typed donor-recipient pairs, the distribution of mismatches (MM) ranged from 3/8 to 8/8: In particular, there 3 cases (5%) with 3/8 MM; 5 cases (8%) with 4/8 MM; 10 cases (16%) with 5/8 MM; 12 cases (20%) with 6/8 MM; 20 cases (32%) with 7/8 MM; 12 cases (20%) with 8/8 MM. These results show that a high degree of HLA MM (7/8 or 8/8) was present in more than 50% of the overall cohort of transplanted patients. In addition, when comparing the populations of transplanted patients with or without DSA, in both populations we observed a high degree of HLA MM (7/8 and 8/8) in 60% and 51% of cases, respectively. The small sample size, however, did not enable statistical evaluations.

### 3.2 High plasma dd-cfDNA levels in recipients with immunological complications (*Group 2*)

A total of 127 dd-cfDNA levels were measured in the 100 patients with median plasma dd-cfDNA level of 0.58% (IQR 0.29%–1.65%). Forty-six percent of samples exceeded the clinically relevant threshold of 1%. The dd-cfDNA levels in plasma, assessed across different patient groups, showed a clear association with graft injury. Plasma dd- cfDNA levels were significantly higher in patients with graft injury, with a median of 1.27% (IQR 0.49%–3.65%) compared to 0.39% (IQR 0.22%–0.61%) in those without graft injury (p < 0.001). Stratifying by injury type, patients with immunological injury (*Group 2*) exhibited the highest plasma dd-cfDNA levels, with a median of 2.67% (IQR 1.38%–4.06%), while non-immunological cases showed lower levels (median 0.68%, IQR 0.34%–1.15%). In *Group 2*, the highest median values of dd-cfDNA were observed in AMR cases (median: 2.77%, IQR 1.25%–4.9%). Patients with both immunologic and non-immunologic injuries exhibited the highest median plasma dd-cfDNA levels, with a median of 3.39% (IQR 1.39%–3.93%) (**Table 2**).

The ROC curve of *Group 2* showed an area under the curve (AUC) AUC of 0.93 (95% CI 0.88-0.99) and a sensitivity of 100% (positive predictive value - PPV= 71%, negative predictive value - NPV=100%) and overall accuracy of 90% (p = 0.003). In contrast, the ROC curve of *Group 3* showed an AUC of 0.63 (95%CI 0.50-0.76) and a much lower sensitivity of 28%, (PPV=57%, NPV=68%) with an overall accuracy of 66%. No difference was observed in terms of percentage level comparing different forms of infections (bacterial, vs viral vs fungal) and infections with other injuries such as lung aspiration. Even insignificant multiple agents (viruses and/or bacterial) showed a higher dd-cfDNA level compared to single agents (median: 2.98, IQR: 0.28-3.35 vs median: 1.12, IQR: 0,29-1.39; p = ns).

**Figure 2:**
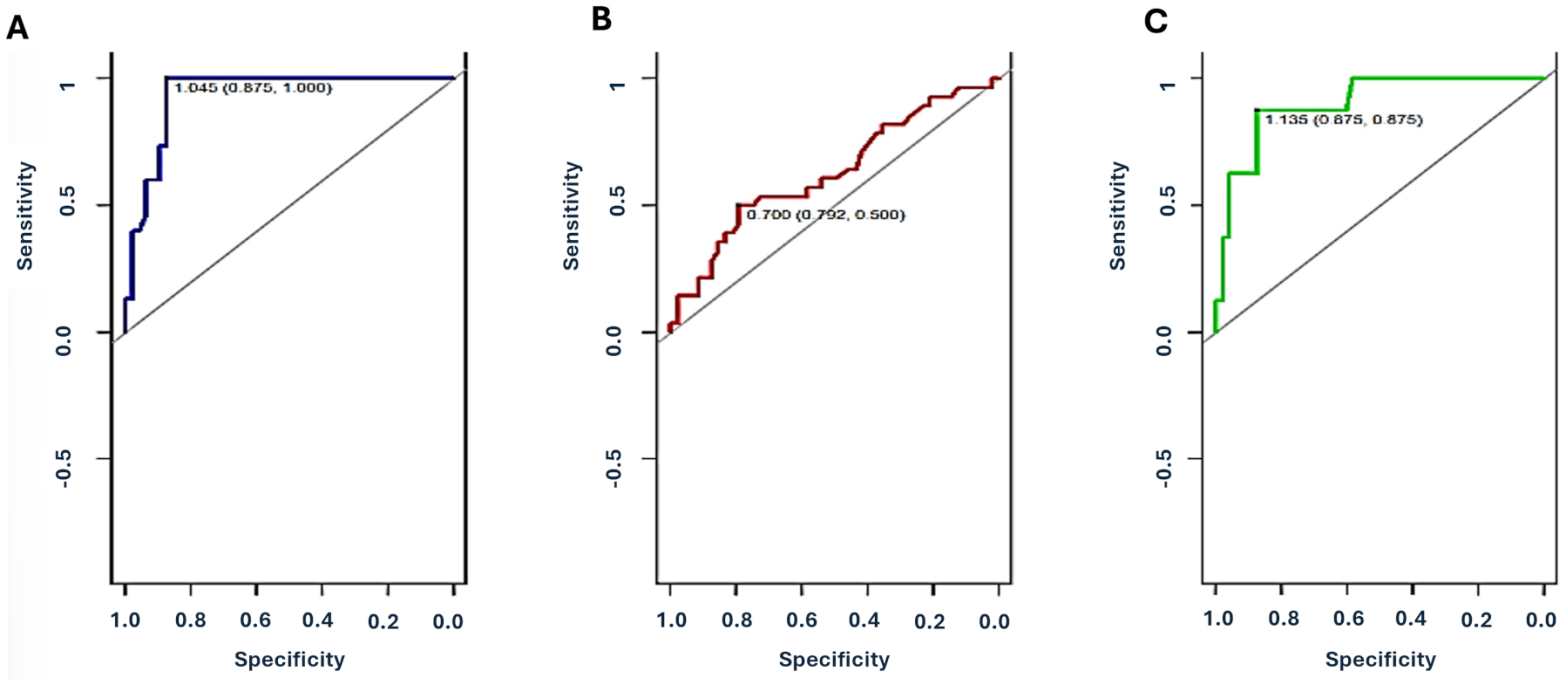
Receiver Operating Characteristic (ROC) curves for plasma dd-cfDNA in different categories of injury. **(A)**: *Group 2* (immunological damage: ACR, AMR and CLAD), **(B)** *Group 3* (non-immunological damage: infections, GER-lung aspiration), **(C)** *Group 4* (both immunological and non-immunological injuries). ROC-AUC were 0.93 (95%CI 0.88-0.99) for *Group 2*, 0.63 (95%CI 0.50-0.76) for *Group 3*, and 0.90 (95%CI 0.79-1.0) for *Group 4*. Values reported in the figures are the optimal cutoff and respective 95% CI for each injury category.

The ROC curve for patients of *Group 4* showed an AUC of 0.90 (95% CI 0.79-1.0), a sensitivity of 87% (PPV=54%, NPV=98%) and accuracy of 88% **Figure 2**, **Table 3**.

**Table 3:** Diagnostic performance for plasma dd-cfDNA by injury type.

Injury type	Threshold [median (CI 95%)	Sensitivity	Specificity	PPV	NPV	Overall accuracy
Immunological injury	1.045 (0.870,1)	100	87	71	100	90
Non-immunological injury	0.7 (0.702,0.5)	28	87	57	67	65
Mixed immunological and non-immunological injury	1.135 (0.875,0.875)	87	87	53	97	87

PPV, positive predictive value; NPV, negative predictive value. CI, confidence interval.

**Figure 3:**
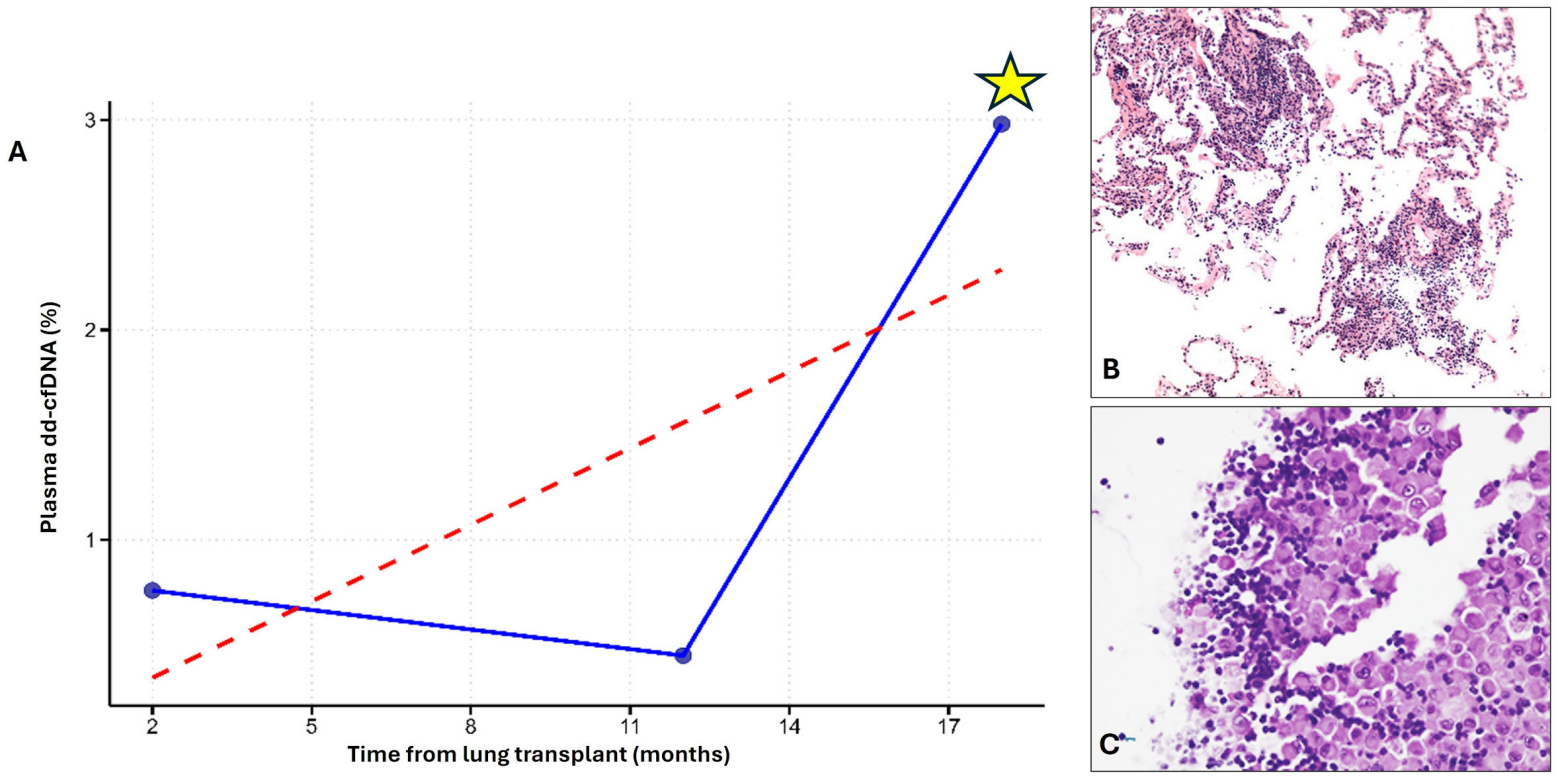
Explanatory case with 3-time plasma dd-cfDNA evaluations in the surveillance follow-up. A) Longitudinal plot showing three timepoints of plasma dd-cfDNA measurements. A marked peak in plasma dd-cfDNA (yellow asterisk) exceeded the 1% threshold (indicated by the red dashed line) and coincided with a period of significant graft injury **(A)**. Transbronchial biopsy performed at this time revealed A3Bx acute cellular rejection, confirming immune-mediated injury **(B)**. BAL analysis from the same timepoint revealed multiple viral infections and lymphocytosis, consistent with concomitant non-immune injury **(C)** (Red Dashed Line: 1% Plasma dd-cfDNA Threshold; Blue Line: Patient’s Longitudinal dd-cfDNA).

Twenty-seven serial plasma samples were obtained from patients with more than two assessments. These follow-up samples were collected during scheduled surveillance visits as part of routine post-transplant monitoring and were not specifically driven by rejection episodes, non-response to therapy, or persistent dysfunction. In general, in our center, if an episode of ACR is diagnosed, patients are treated and re-evaluated after 40 days with repeat transbronchial biopsies and pulmonary function tests. If histological signs of rejection persist, a second course of high-dose steroids is administered. Should the rejection fail to fully regress after an additional 40 days, and after ruling out other potential causes of non-response such as Cytomegalovirus infection or subtherapeutic immunosuppressant levels, a change in maintenance immunosuppressive regimen is considered. Of these recipients only one showed a severe graft injury (concomitant A3Bx + multiple viral infections) at the last surveillance follow-up: at this time a high dd-cfDNA level (2.98) was detected **Figure 3**. This patient experienced a bacterial/fungal infection with an associated mild sequalae of ischemia/reperfusion injury scored by LASHA ≤2 (dd-cfDNA: 0.76), which resolved by the time of the second scheduled visit (dd-cfDNA: 0.45).

Univariate analysis showed that time from lung transplantation, native diseases other than obstructive and restrictive forms, mainly including idiopathic pulmonary hypertension, and donor age were associated with higher dd-cfDNA values (p < 0.0001, p = 0.024, p = 0.03, respectively) (**Supplementary Table 2**). However, after the multivariate regression analysis the only parameter associated with increased dd- cfDNA levels (Beta = 0.02, 95% CI [0.01, 0.03], p < 0.001), without any difference among groups was the time from lung transplantation (**Supplementary Table 3**). All other donor or recipient characteristics showed no significant influence on dd- cfDNA levels in all groups.

### 3.3 Low performance of BAL for allograft injury detection

A total of 60 BAL dd-cfDNA levels were measured in 60 patients with concomitant plasma evaluation. Four samples were not processed due to inadequate DNA. The cut-off of BAL was 10%. The dd-cfDNA levels in BAL fluid were substantially higher than those in plasma, with a median of 12% (IQR 5%–23%). Unlike plasma dd-cfDNA, BAL dd-cfDNA levels did not significantly differentiate between patients with and without graft injury (p = 0.060).

After stratification by injury type, *Group 3* patients showed a better sensitivity than plasma dd-cfDNA (43% vs 28%), even if low specificity (**Supplementary Table 4**). The cutoff for multiple infections (bacterial and viral) was 16.17% (IQR 12–20), significantly higher than that for single infections, which was 1.88% (IQR 0.7–1.9) (P = 0.037). Aspiration did not influence the significance level.

### 3.4 Combined plasma and BAL analysis

All performance metrics presented in this section refer exclusively to the 60 patients who had paired plasma and BAL sampling. Within this subcohort, the performance of plasma dd-cfDNA alone for detecting non-immunological injury mirrored the results from the larger 100-patient cohort, with a sensitivity of 28%. The addition of BAL dd-cfDNA (using a 10% cut-off) improved sensitivity for detecting non-immunologic injury to 71%, recovering 4 out of 8 cases that plasma alone had missed. Importantly, this diagnostic gain was limited to Group 3 (infections or aspiration-related injuries); there was no added benefit observed in Group 2 (immunological) or Group 4 (mixed) injuries. Thus, whilst adding BAL had no value in the case of immune-mediated damage, our findings demonstrate a clear complementary role of BAL dd-cfDNA in non-immunological cases.

Additionally, we evaluated the performance of the combined molecular rule (plasma >1% or BAL ≥10%) against histological outcome. The combined dd-cfDNA approach showed a sensitivity of 65% (95% CI: 42–82%), specificity of 85% (63–96%), PPV of 83% (60–95%), NPV of 68% (45–86%), and overall accuracy of 74% (59–86%). The molecular assay missed 35% of biopsy-positive cases.

### 3.5 Variables importance

**Figure 4:**
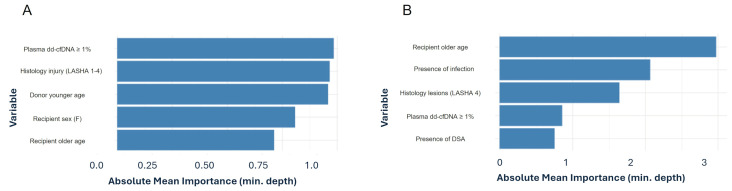
Variable importance of the top five predictors derived from random forest classifying for death **(A)** and CLAD **(B)**.

The Cox proportional hazards regression identified two significant predictors of adverse outcomes (development of CLAD or death), in LT recipients: older recipient age and chronic rejection-associated lesions described by LASHA. Recipient age was associated with a 5% increased risk of adverse events for each additional year (Hazard ratio - HR = 1.0467, 95% CI: 1.0075–1.087, p = 0.019). Suggestive histological lesions of chronic rejection increased the risk of CLAD development (HR = 6.50, 95% CI: 2.69–156.9, p = 0.0102) and in general of adverse outcome (HR = 3.48, 95% CI: 1.03–11.7, p = 0.044). Random Forest survival analysis showed that plasma dd-cfDNA higher than 1% was among the most important variables in influencing CLAD and death. In addition, we assessed the direct association between plasma dd-cfDNA levels and CLAD using a univariable logistic regression model. Applying the pre-specified 1% plasma dd-cfDNA threshold, recipients with values above this cut-off had approximately 16 times higher odds of being diagnosed with CLAD compared to those below it (Odds Ratio = 16.0, 95% CI = 2.6–305, p = 0.012). Although the confidence interval is wide due to the limited number of CLAD cases (n = 10), the lower bound of the interval remains well above 1, indicating a statistically meaningful association between elevated plasma dd-cfDNA and CLAD status. (**Figures 4A, B**).

## 4 Discussion

In this monocentric, prospective cohort study we found that following lung transplantation plasma dd-cfDNA is a highly sensitive and specific biomarker for detecting immunological events related to both acute and chronic graft rejection. To our knowledge this is the first study to evaluate plasma dd-cfDNA levels in different post-transplant scenarios using a multidisciplinary integrated approach. Our methodology incorporated systematic TBB by LASHA and analytic cellular evaluation of BAL, in addition to several post-transplant routine follow-up tests. Additionally, we explored for the first time the potential added value of BAL dd-cfDNA measurements alone and in combination with plasma dd-cfDNA.

Given the complexity of lung transplant recipient management, and the frequency of both immunological and non-immunological complications which may occur, a multidisciplinary approach is increasingly becoming standard of care (15). Indeed, lung transplant recipients are at continued risk of infection which remains a leading cause of morbidity and mortality beyond the first-year post transplantation (16). MDT discussions are crucial in this setting, where differentiating between infection and rejection is often challenging due to overlapping clinical and histological features. Several international statements/guidelines (11, 12, 17),have clearly highlighted the importance of obtaining a clear definition of post-transplant complications (14, 17–19). Our study prioritized this aspect by evaluating the diagnostic value of plasma and BAL dd-cfDNA in real-world practice, integrating them with established diagnostic tools.

We observed a high sensitivity and specificity (100% and 87%, respectively) of plasma dd-cf DNA in detecting immunological events. Elevated dd-cfDNA levels were primarily found in AMR cases where TBBs demonstrated moderate to severe septal neutrophilic infiltration- a hallmark AMR lesion in LASHA. Previous studies have reported increased plasma dd-cfDNA levels in lung transplant recipients with immunological disorders (2, 4, 6, 20) with dd-cfDNA levels consistent with our findings. AMR diagnosis remains challenging despite the contribution of a multidisciplinary assessment. The addition of dd-cfDNA could enhance diagnostic confidence as demonstrated in **Table 4** and in **Supplementary Figure 2** (11, 21 **).**

**Table 4:** Clinical scenarios with suspected AMR and the added value of dd-cfDNA.

Case	Clinical & Histological Findings	Initial Amr Classification (Ishlt 2016)	dd-cfDNA Result	Revised Diagnostic Confidence	Potential Clinical Impact
1	Histological suspicion; DSA+; borderline BAL findings	Possible/Clinical AMR	Elevated (>1%)	Clinical Probable AMR	Supports closer monitoring or treatment initiation
2	Subclinical DSA+; mild septal infiltration	Subclinical/Possible AMR	Borderline (≈1%)	Clinical Possible AMR	May support further immunologic workup
3	Infectious signs; no DSA; no histological AMR lesions	No AMR	Low (<1%)	AMR unlikely	Reinforces infection as primary cause; avoid overtreatment

Interestingly, plasma dd-cfDNA levels were also increased in patients with coexistent immunological and non-immunological injuries (*Group 4)*, suggesting that more extensive graft damage leads to increased donor DNA release.

Multivariate analyses revealed that episodes of elevated plasma dd-cfDNA were time-matched. Previous reports (5, 22, 23) showed a gradual increase in dd-cfDNA (%) over time, thought to be due to the influence of immunosuppressive treatments leading to alterations of white blood cells. The decrease of total cfDNA over time may result in apparently elevated dd-cfDNA percentage values. The detected dd-cfDNA time dependent rate was however minimal (0.02%) and substantially similar in all groups reinforcing that elevated dd-cfDNA primarily reflects graft injury severity.

Although follow-up sampling was not systematically designed to assess post-treatment response, we analyzed 27 longitudinal samples obtained during routine surveillance visits, and only one patient showed a significant dd-cfDNA peak (2.98%) coinciding with combined immunological and non-immunological injury. In contrast, when infection was detected, the dd-cfDNA level (occurred 12 months after lung transplantation) was below the cut off (**Figure 3**). While limited by sample size, this observation supports the potential value of serial dd-cfDNA monitoring in identifying subclinical or evolving graft dysfunction. Importantly, the dd-cfDNA measurements included in our study were all obtained during routine surveillance visits, not during episodes of clinically apparent graft dysfunction. This design provides a real-world estimate of the performance of dd-cfDNA in a stable, asymptomatic post-transplant population. The identification of patients with elevated dd-cfDNA levels who were subsequently diagnosed with graft injury supports the potential of this biomarker for detecting subclinical injury. Furthermore, as the dataset derives exclusively from surveillance procedures, the prevalence of graft injuries reported in this study reflects the baseline risk in a clinically stable cohort, which is essential when interpreting the sensitivity, specificity, and predictive values of dd-cfDNA in this context.

Indeed, in our case series we did not observe elevated plasma dd-cfDNA levels in cases of non-immunological injury such as GER-related lung aspiration or infection, regardless of pathogen type. The addition of dd-cfDNA as an indicator in such contexts remains debated in the literature (7). Bazemore et al. reported a correlation between plasma dd-cfDNA levels and specific infectious pathogens at risk of graft progression (8, 9). However, due to our small sample size, we were unable to analyze pathogen categories individually, especially high-risk fungi. The low rate of fungal infections may also reflect the use of prophylactic azoles in the post-operative period, which likely reduced the occurrence of fungal infections in our cohort.

Exploratory BAL ddcf-DNA analyses revealed consistently higher levels than in plasma, suggesting increased DNA release from injured lung tissue into BAL fluid.

Only two preliminary studies about BAL dd-cfDNA detected specifically by using NGS have been reported, finding discrepancies about its specificity. The authors reported doubtful results on the true origin of cfDNA content (recipient rather than donor) and non-perfect matches with the plasma dd-cfDNA levels ​​in different graft injuries, respectively . (11, 21), Our inclusion of pre-and post-transplant BAL cfDNA isolated from plasma and BAL reference samples confirmed that donor DNA constituted the majority of the BAL sample, supporting its potential as a lung specific injury marker.

The higher BAL dd-cfDNA content detected in our series may suggest that more DNA is released from the lung into the BAL than into the bloodstream, making BAL potentially a more effective tool for detecting injuries occurring within the graft. Similarly, studies comparing dd-cfDNA levels in urine and blood in kidney transplantation have suggested that locally sourced samples, such as urine (24–26 ^)^ may exhibit elevated but variable sensitivity for detecting injuries confined to the organ of origin. However, the reasons for these inconsistencies remain unclear, as high sensitivity does not always correlate with accurate or reliable performance. These findings underscore the need for additional experimental and clinical studies to better understand and optimize the use of locally sourced samples, such as BAL fluid, as indicators of lung-specific injuries.

Although the BAL dd-cfDNA performance in detecting graft injury was generally limited, its value was particularly evident in the non-immunological group. In the subset of 60 patients with paired samples, BAL dd-cfDNA alone demonstrated higher sensitivity than plasma (43% vs 28%). Furthermore, when plasma and BAL analyses were combined, sensitivity for detecting non-immunological injuries rose to 71%, with 4 out of 8 plasma-negative cases being correctly reclassified. These findings clearly support the incremental diagnostic utility of BAL dd-cfDNA for infections and aspiration-related injury. In contrast, no such benefit was observed in patients with immune-mediated injuries, reinforcing its specific role in non-immune contexts.

Our findings underscore the complementary diagnostic value of BAL dd-cfDNA in conjunction with plasma dd-cfDNA, particularly for non-immune mediated injuries. While plasma dd-cfDNA demonstrated high sensitivity for immune-related graft injury, including ACR, AMR, and CLAD, it showed limited performance in detecting non-immune etiologies such as infection, GERD, or aspiration. Importantly, BAL dd-cfDNA was able to identify several cases of injury that were not detected by plasma dd-cfDNA alone, particularly in the context of infectious or aspiration-related injury. This result supports the utility of BAL dd-cfDNA not only as an adjunct to plasma analysis but also as a potential discriminator between true infection and microbial colonization. These findings reinforce the notion that dual-compartment analysis may enhance diagnostic accuracy and clinical decision-making in the management of lung allograft dysfunction. While the combined plasma and BAL dd-cfDNA approach offered high specificity and positive predictive value for biopsy-confirmed graft injury, its lower sensitivity indicates that molecular testing alone is not yet sufficient to replace histopathological evaluation. These results highlight the need for an integrated diagnostic framework that incorporates plasma dd-cfDNA alongside established modalities such as histopathology, DSA assessment, imaging, and clinical evaluation. Given its high sensitivity for detecting immunologic injury and limited utility in non-immunologic contexts, plasma dd-cfDNA should be interpreted within the broader clinical context.

This work has several limitations including its observational nature and a single center design. While unicentric, the study benefits from having all patients managed under a uniform immunosuppressive approach and a standardized lung monitoring and sampling methodology. Furthermore, we are strongly confident that we have reported a more precise category of post-transplant complications derived from the MDT discussion.

Another relevant limitation concerns the pre-analytical handling of BAL samples: although all samples in this study were processed within a 2-hour window, such conditions may not be consistently achievable in real-world clinical practice. This could limit the widespread applicability of BAL dd-cfDNA analysis outside of controlled research environments. In high-volume transplant centers, implementing standardized operating procedures and allocating dedicated personnel may be necessary to support timely sample processing and enable the clinical adoption of this diagnostic tool.

The cohort included relatively few ACR episodes due to a median post-transplant time of ten months, but the study’s real-world design ensured consecutive enrollment of all patients undergoing scheduled clinical visits over a two-year period. Additionally, we acknowledge that the availability of immunological data was incomplete in a subset of patients. The limited availability of these data also impacts the interpretation of metrics such as sensitivity, specificity, PPV, and NPV, which are inherently influenced by disease prevalence and immunological risk. However, our statistical analyses accounted for the limited sample size and multiple covariates, though some risks of the estimate instability remain.

In conclusion, plasma dd-cfDNA is significantly elevated in lung transplant recipients with immunological disorders particularly in patients with AMR. While BAL dd-cfDNA alone showed limited diagnostic value across all injury types, its combination with plasma dd-cfDNA significantly improved detection of non-immunological injuries within the subgroup of patients with paired sampling. These results suggest that incorporating BAL dd-cfDNA into standard surveillance, particularly for recipients at higher risk of infection or aspiration, may enhance diagnostic precision and support more tailored post-transplant care.

## Data Availability

The raw data supporting the conclusions of this article will be made available by the authors, without undue reservation.
