# Supplementary material for: Detection of lung allograft injury through a comprehensive multidisciplinary analysis of donor-derived cell-free DNA in plasma and bronchoalveolar lavage: a real-world single center experience
Source: Front Immunol. 2025 Sep 5;16:1619771. doi: 10.3389/fimmu.2025.1619771 (PMC12447569; doi:10.3389/fimmu.2025.1619771)

Supplementary Material

# Supplementary Data

**1.1 Patient clinical data**

Data on the study population, including demographic information, the primary indication for lung transplantation, donor/recipient characteristic, all morphological/humoral, microbiological, clinical/functional data at time of scheduled visit and last follow-up were recorded in a dedicated database**.** Allograft dysfunction was defined as a decline in FEV1 (forced expiratory volume in the first second) ≥ 10% and was categorized as “no” (< 10%), “mild” (≥ 10% to < 15%), or “moderate to severe” (≥ 15%) allograft dysfunction as previously described. (1)

CLAD was defined using the International Society for Heart & Lung transplantation criteria.(2) Patients were further classified as having obstructive syndrome (bronchiolitis obliterative syndrome) or restrictive syndrome (restrictive allograft syndrome) after multi-disciplinary team discussion taking into consideration PFT, computed tomography scan and/or histology (see below).(3)

All lung transplant recipients received induction therapy with basiliximab combined with a triple-agent immunosuppressive regimen including tacrolimus, mycophenolate mofetil, and methylprednisolone. Cell-mediated rejection events established by histology and examination were mainly treated with pulse dose glucocorticoids (methylprednisolone 15 mg/kg/day for 3 days). In our cohort 17% of patients had previous immunologic insults, mild/moderate ACR (A2 and A3) successfully treated with immunosuppressive therapy and 4% of them possible subclinical AMR (only DSA positive; not treated and only managed with a strict follow-up), as retrieved from the records. Antimicrobial prophylaxis was administered following our institutional protocol.

**1.2 Microbiological and DSA screening**

Molecular analyses involved the use of the MagNA Pure Compact Nucleic Isolation Kit I (Roche Molecular Systems, California, United States) to extract both DNA and RNA in accordance with the manufacturer's instructions. The following viral or bacterial microorganisms were investigated using in-house RT-PCR 7900HT RT-PCR System (Applied Biosystems, California, United States) validated for clinical use (Chlamydophila pneumonia, Herpes virus simplex (HSV) types 1 and 2, Epstein-Barr virus (EBV), Adenovirus, Mycoplasma pneumoniae, Bordatella pertussis, Cytomegalovirus (CMV), Coronavirus HKU1, Coronavirus NL63, Coronavirus UPE, Coronavirus 229, Coronavirus OC43, Parainfluenza virus types 1, 2, and 3, Influenza viruses A and B, Rhinovirus, and respiratory syncytial virus).

**1.3 Histopathology and cytological analysis**

Mild histological injuries scoring 1 on the LASHA scale and limited to the alveolar septa or interalveolar compartment were considered unremarkable for defining injury particularly in the absence of microbiological or immunological abnormalities. Using the LASHA template histological signs evocative of AMR were evaluated and graded in different anatomical compartments which included septal neutrophils, edema or widening, septal/intra-alveolar neofibrogenesis (OP), intra-alveolar hyaline membrane, pneumocyte hyperplasia, and the presence of acute fibrinous and organizing pneumonia (AFOP).

Histological signs suggestive of chronic rejection were also systematically assessed according to the LASHA template and these which included along obliterative bronchiolitis (OB) indirect signs of OB such as mucostasis, intra-alveolar foamy macrophages, bronchiectasis and evocative lesions of RAS such as septal fibrous NSIP-like thickening and pleural parenchymal fibroelastosis (rarely occurring in peripheral lung parenchyma samples). Histological signs of obliterative bronchiolitis (grade C) suggestive of chronic rejection or indirect signs of obstruction or restrictive allograft syndrome (RAS) were noted and discussed in multidisciplinary meetings to assure accurate categorization. Special stains for microorganisms were also used, although a more sensitive assessment of infection was performed on BAL collected simultaneously.

BAL was performed with a fiberoptic bronchoscope in a wedge position within the selected broncho-pulmonary segment. The total instilled volume of normal saline was ≥ 100 ml and < 300 ml. For an optimal sampling of distal airspaces, the total retrieved volume (pooled aliquots) was kept ≥ 30% of the total instilled. Finally, the total retrieved volume was divided into two aliquots (usually of 10-15 ml), one of which was used for microbiological assays, and the other was stored for cytological analyses. At least four slides were stained with Giemsa, Papanicolaou, Gram, and Gomori-methenamine-silver, with the last two being used for bacterial and fungal element detection. Oil red staining was also carried out as useful tool for verifying the diagnosis of lung aspiration. The lipid laden macrophage index was calculated using the method proposed by Colombo and Hallberg.(4)

BAL cytological analyses included the evaluation of the following parameters: (i) BAL adequacy and representativeness, when the percentage of ciliated columnar cells was < 5% in the quantification of inflammatory cellular component, (iii) identification of microorganisms with appropriate special stains, and (iv) description of other components (fibrin, pneumocytes, blood). (5)

**1.4 Detection of dd-cfDNA in plasma samples and BAL**

1.4.1 Sample Collection and Processing

All plasma and BAL samples were collected in Streck Cell-Free DNA BCT® tubes (Streck LLC, NE, USA) and processed within two hours of collection. Following centrifugation to isolate the cell-free fraction, samples were aliquoted and stored at −80°C.

1.4.2 cfDNA Extraction and Library Preparation

Extraction of cfDNA from plasma and BAL supernatants was performed using the QIAamp MinElute ccfDNA Kit (QIAGEN, Venlo, Netherlands), according to the manufacturer’s protocol. Library construction was completed using the AlloSeq® cfDNA kit (CareDx, CA, USA), designed for high-sensitivity quantification of dd-cfDNA via single-nucleotide polymorphism (SNP) discrimination.

1.4.3 Next-Generation Sequencing (NGS) and SNP Analysis

Libraries were sequenced on the Illumina Miniseq platform (2 × 150 bp paired-end reads). SNP genotyping was conducted by comparing donor and recipient genomic DNA, enabling quantification of dd-cfDNA as a percentage of donor-specific alleles among total SNP reads. Only high-quality reads meeting predefined QC metrics were included. Replicate analyses were performed for reproducibility by two independent operators.

1.4.4 Cut-off Thresholds and Validation

Plasma dd-cfDNA cut-off: A threshold of 1% was used, as predefined by the manufacturer (CareDx) and supported by previously published validation studies in solid organ transplantation. (6,7)

BAL dd-cfDNA cut-off: A threshold of 10% was empirically derived from internal ROC curve optimization using a small pre-/post-transplant control group (n = 20) and further cross-validated in the study cohort. This threshold demonstrated acceptable sensitivity for non-immunologic injuries and was supported by SNP-matched genotyping to confirm donor origin of BAL cfDNA.

**2. Data integration and statistical analysis**

*ROC curves and cut-offs*

Specifically, 10-fold cross-validation was applied using the caret package in R. The entire dataset was randomly partitioned into ten folds of approximately equal size, stratified by injury category to preserve the minority prevalence. For each iteration, a model was fitted on nine folds and evaluated on the remaining fold. The area under the curve (AUC) reported is the mean of these ten out-of-fold estimates. Max Youden Index was used within the cross-validation to identify the optimal cut-off point.

To quantify uncertainty, 95% confidence intervals (CI) were derived using the DeLong non-parametric method (implemented via pROC::ci.auc), applied to the vector of cross-validated predictions for each sample. The relatively narrow CI for group 2 (0.88–0.99) reflects between-fold variability, thus reducing the risk of optimistic bias despite the limited number of immunological events.

This process was repeated k times, with each fold serving once as the validation set, thus reducing bias and overfitting risks. The final model performance metrics (e.g., sensitivity, specificity, positive predictive value (PPV), negative predictive value (NPV), and accuracy) were averaged across all folds.

*Linear Regression Analysis*

The regression model was fitted using the ordinary least squares (OLS) method. Model assumptions, including linearity, homoscedasticity, and normality of residuals, were evaluated using graphical diagnostics such as residual plots and Q-Q plots. Continuous variables were checked for linear relationships with the outcome, and categorical variables were assessed for multicollinearity.

*Variables selection (****Table S1****)*

- *Model Implementation:* For each outcome, random forest models were trained using the ranger package in R, with survival-specific splitting rules based on the log-rank test.
- *Variable Importance:* Predictor importance was quantified using impurity-corrected measures (minimum depth), which account for potential biases associated with predictor scale and number of categories
- *Stability Across Random Seeds:* To ensure robustness of the findings, we repeated the analysis across five random seeds (1, 13, 135, 3011, 10058). For each seed, the random forest model was refitted, and variable importance metrics were computed. Variables consistently ranking across all seeds were identified as robust predictors. For each outcome, predictors appearing in all five random forest iterations were summarized as importance metrics (mean, minimum, and maximum importance scores were computed for each variable). Variables were ranked based on their absolute mean importance, and results were visualized using bar plots.

All analyses and graphics were done using R (v. 4.4.1) and the packages gtsummary, ranger, caret, and pROC.

**References**

1. Jang MK, Tunc I, Berry GJ, Marboe C, Kong H, Keller MB, et al. Donor-derived cell-free DNA accurately detects acute rejection in lung transplant patients, a multicenter cohort study. The Journal of Heart and Lung Transplantation. 2021 Aug;40(8):822–30.

2. Verleden GM, Glanville AR, Lease ED, Fisher AJ, Calabrese F, Corris PA, et al. Chronic lung allograft dysfunction: Definition, diagnostic criteria, and approaches to treatment―A consensus report from the Pulmonary Council of the ISHLT. The Journal of Heart and Lung Transplantation. 2019 May;38(5):493–503.

3. Lunardi F, Abbrescia DI, Vedovelli L, Pezzuto F, Fortarezza F, Comacchio GM, et al. Molecular Profiling of Tissue Samples with Chronic Rejection from Patients with Chronic Lung Allograft Dysfunction: A Pilot Study in Cystic Fibrosis Patients. Biomolecules. 2023 Jan 3;13(1):97.

4. Colombo JL, Hallberg TK. Recurrent aspiration in children: Lipid‐laden alveolar macrophage quantitation. Pediatr Pulmonol. 1987 Mar 20;3(2):86–9.

5. Calabrese F, Lunardi F, Baldasso E, Pezzuto F, Kilitci A, Olteanu GE, et al. Comprehensive bronchoalveolar lavage characterization in COVID-19 associated acute respiratory distress syndrome patients: a prospective cohort study. Respir Res. 2023 Jun 9;24(1):152.

6. Agbor-Enoh S, Wang Y, Tunc I, Jang MK, Davis A, De Vlaminck I, et al. Donor-derived cell-free DNA predicts allograft failure and mortality after lung transplantation. EBioMedicine. 2019 Feb;40:541–53.

7. Sayah D, Weigt SS, Ramsey A, Ardehali A, Golden J, Ross DJ. Plasma Donor-derived Cell-free DNA Levels Are Increased During Acute Cellular Rejection After Lung Transplant: Pilot Data. Transplant Direct. 2020 Oct;6(10):e608.

# Supplementary Tables

**Supplementary Table 1.** Variables included in the model for variable importance estimation.

| Variable | Description |
| --- | --- |
| Baseline recipient characteristic |  |
| Sex | 1 = Male, 2 = Female |
| Age | Age in years |
| Native disease | Type based on lung dysfunction (1 = Restrictive, 2 = Obstructive, 3 = Other) |
| Baseline donor characteristic |  |
| Sex | 1 = Male, 2 = Female |
| Age | Age (years) |
| Cause of death | Stroke/intracranial hemorrhage=1, traumatic brain injury=2; anoxia= 3, cardiovascular (DCD)=4, others=5 |
| Expanded donor criteria | Marginal donors: 1=Yes, 2=No |
| Time of biopsy | Time from transplant to biopsy (months) |
| At time of biopsy |  |
| LASHA* | LASHA 1: unspecific histological injuries; LASHA 2: histological lesions suggestive of infection: intra-alveolar neutrophils/organizing pneumonia/airway inflammation; LASHA 3: acute cellular rejection; LASHA 4: obliterative bronchiolitis and other signs suggestive of chronic rejection; LASHA 5: suggestive lesions of antibody mediated rejection: septal neutrophils/hyaline membrane/organizing pneumonia |
| Clinical/Functional parameter | Symptoms/sign of acute dysfunction: 1= Yes; 2=No  Symptoms/signs of chronic dysfunction-CLAD-: 1: yes; 2: no |
| BAL* cytology | Cell phenotype: 0=normal; 1=neutrophilia; 2= lymphocytosis |
| BAL microbiology | Detection of infection 1 = Yes, 2 = No |
| Plasma dd-cfDNA value | cfDNA percentage in blood (cut-off: 1%) |

_* Abbreviations: LASHA (lung allograft standardized histological analysis), BAL (bronchoalveolar lavage)_

**Supplementary Table 2.** Univariate analysis comparing plasma dd-cfDNA levels with the most significant baseline recipient/donor characteristic data and time of LTX

| Variable | Beta | 95% CI* | p-value |
| --- | --- | --- | --- |
| Time from lung transplant (months) | 0.02 | 0.01-0.03 | < 0.001 |
| Native disease |  |  |  |
| *Restrictive* | - | - |  |
| *Obstructive* | 0.61 | -0.23-1.5 | 0.2 |
| *Other* | 1.2 | 0.17-2.3 | 0.024 |
| Recipient age | -0.02 | -0.05-0.00 | 0.089 |
| Recipient sex |  |  |  |
| *Males* | - | - |  |
| *Females* | 0.08 | -0.73-0.89 | 0.8 |
| Donor age | -0.03 | -0.05-0.00 | 0.03 |
| Donor sex |  |  |  |
| *Males* | - | - | - |
| *Females* | -0.32 | -1.1-0.48 | 0.4 |
| Expanded**** criteria donor |  |  |  |
| *Yes* | - | - | - |
| *No* | 0.72 | -0.07-1.5 | 0.074 |

**Confidence interval*

**Supplementary Table 3.** Multivariate regression analysis comparing plasma dd-cfDNA levels with most significant baseline recipient/donor characteristic data and time of LTX

| Variable | Beta | 95% CI* | p-value |
| --- | --- | --- | --- |
| Time from lung transplant (months) | 0.02 | 0.01- 0.03 | < 0.001 |
| Native disease |  |  |  |
| *Restrictive* | - | - | - |
| *Obstructive* | -0.07 | -0.95- 0.82 | 0.9 |
| *Other* | 0.90 | -0.11- 1.9 | 0.081 |
| Recipient age | -0.01 | -0.04- 0.02 | 0.5 |
| Recipient sex |  |  |  |
| *Males* | - | - | - |
| *Females* | -0.18 | -0.93-0.57 | 0.6 |
| Donor age | 0.00 | -0.03- 0.04 | >0.9 |
| Donor sex |  |  |  |
| *Males* | - | - | - |
| *Females* | -0.27 | -1.1-0.59 | 0.5 |
| Expanded**** criteria donor |  |  |  |
| *Yes* | - | - | - |
| *No* | 0.04 | -0.91-0.98 | >0.9 |

**Confidence interval*

**Supplementary Table 4.** **Diagnostic performance for BAL dd-cfDNA by injury type**

| **Injury type** | **Sensitivity** | **Specificity** | **PPV** | **NPV** | **Overall accuracy** |
| --- | --- | --- | --- | --- | --- |
| **BAL cfDNA** |  |  |  |  |  |
| **Group 2*** | 20 | 48 | 4 | 82 | 45 |
| **Group 3**** | 42 | 50 | 28 | 65 | 47 |
| **Group 4***** | 40 | 46 | 86 | 85 | 45 |

*PPV: positive predictive value; NPV: negative predictive value*

** Group 2: patients with immunological damage: ACR, AMR, and CLAD*

*** Group 3: patients without immunological damage with infections or GERD-lung aspiration*

**** Group 4: patients with both immunological and non-immunological damage*

## Supplementary Figures

**Supplementary Figure 1.** Kaplan-Meier survival analysis based on dd-cfDNA positivity.


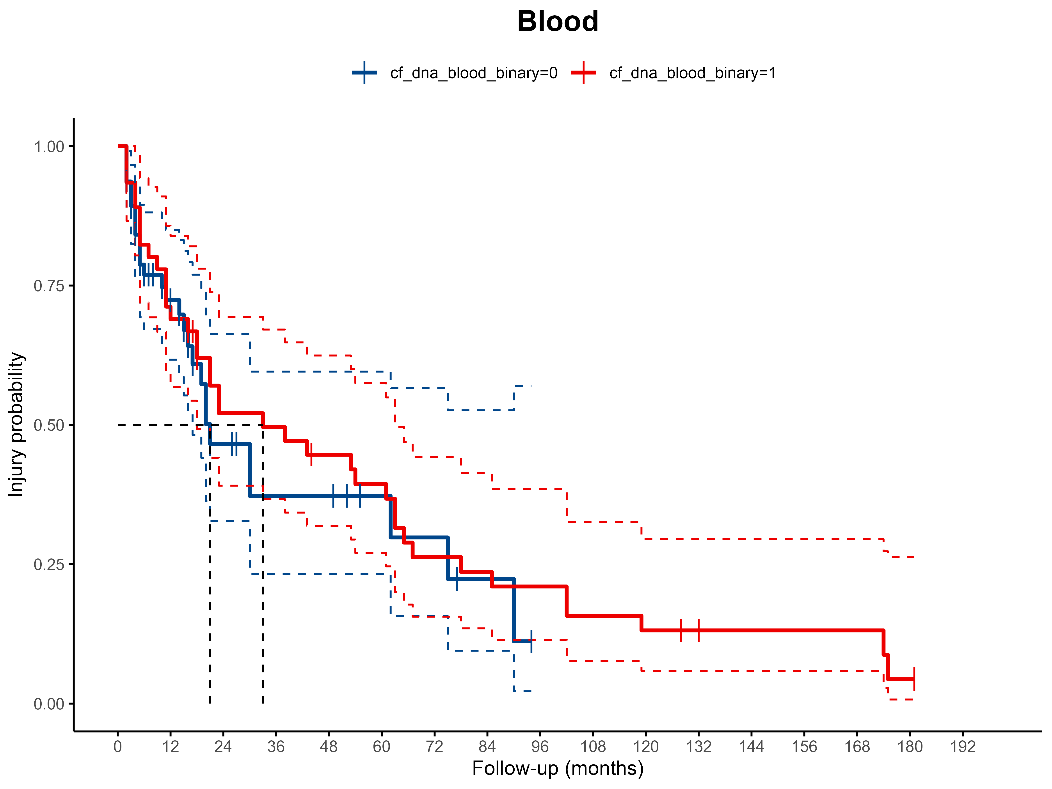


**Supplementary Figure 2.** Clinical case scenarios of suspected lung AMR: In all three cases, including dd-cfDNA would improve the confidence of lung AMR diagnosis. In agreement with 2016 lung AMR consensus statement the diagnosis of definite AMR would not have been made in any case, but it would have been made based on the current statement: Case 1 should be clinical probable AMR; Case 2 subclinical/clinical possible; Case 3: no AMR (infection?).


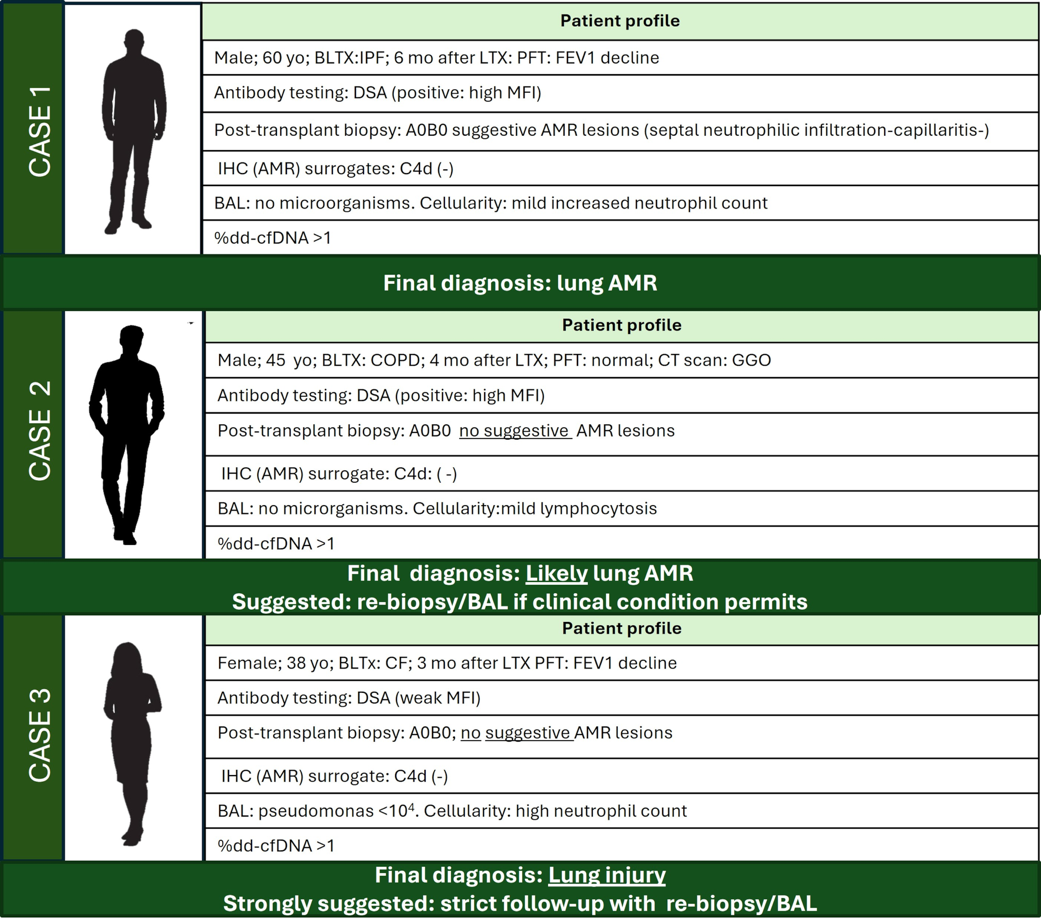

Supplement: Supplementary file 1 [file DataSheet1.docx]
